# In-situ Observation of Hierarchical Self-Assembly Driven by Bicontinuous Gelation in Mixed Nanodisc Dispersions

**DOI:** 10.1038/s41598-018-23814-4

**Published:** 2018-04-03

**Authors:** Ravi Kumar Pujala, C. T. W. M. Schneijdenberg, Alfons van Blaaderen, H. B. Bohidar

**Affiliations:** 1 Soft Condensed Matter group, Debye Institute for Nanomaterials Science, Utrecht University, Princetonplein 5, 3584 CC Utrecht, The Netherlands; 2 School of Physics, University of Hyderabad, Hyderabad, 500046 India; 3 Polymer and Biophysics Laboratory, School of Physical Sciences, Jawaharlal Nehru University, New Delhi, 110067 India; 4 Special Centre for Nanosciences, Jawaharlal Nehru University, New Delhi, 110067 India

## Abstract

The search for new functional soft materials with precise and reconfigurable structures at the nano and meso-scale is a major challenge as well as objective of the current science. Patchy colloids of different shapes and functionalities are considered important new building blocks of a bottom-up approach towards rational design of new soft materials largely governed by anisotropic interactions. Herein, we investigate the self-assembly, growth of hierarchical microstructures and aging dynamics of 2D nano-platelets of two different aspect ratios (Laponite ~25 and Montmorillonite ~250) which form gels with different porosity that is achieved by tuning their mixing ratios. Qualitative *in situ* real-space studies are carried out, including fluorescent confocal microscopy imaging of the bicontinuous gelation process or final states, which provides dynamic visualization of the self-organization. The bicontinuous gels exhibit a foam-like morphology having pores of a few micrometers in size that can be tuned by varying the mixing ratio of nanoplatelets. It is shown that this new class of clay gels has unique and tunable physical properties that will find potential applications in the development of low cost lithium ion batteries, nanocomposites and nuclear waste management.

## Introduction

The whole colloidal length scale, which spans from several nano to micrometres can be used to engineer properties of soft materials. The bottom up fabrication of such materials often relies on hierarchical self-assembly that can be made to follow a well-defined kinetic growth path. Such paths can be achieved by controlling the interactions between the nano-sized building blocks that will in this study be comprised of platelets of various aspect ratios. Making these building blocks geometrically and electrically anisotropic, for example, by tailoring their shape and patchiness will result in the formation of new soft materials with remarkable properties. Colloidal systems can be made to mimic atomic system due to the possibility to control their interaction strengths on several length scales^1^. The self-assembly of colloidal particles with inhomogeneous charge distributions often exhibits anisotropic pair-wise interactions that may yield many phases and states that may be close to or away from thermodynamic equilibrium^2–6^. Nanoclays are ubiquitous in nature and their dispersions are characterized by a rich dynamics. Easy availability coupled with anisotropic interactions has facilitated wide spread use of these materials in the design of nanocomposites, biocompatible products and drug delivery materials^7–10^. As far as the solvation dynamics and phase transition kinetics are concerned, the phenomenon of aging in clay dispersions is still poorly understood, and requires and deeper investigations.

Aging is defined as the change in the physical properties of a system with waiting time that may spread over a period of days to years. The slow dynamics that drives aging owes its origin to the spontaneous lowering of the free-energy of systems which have fallen out of the equilibrium, but still may display dynamics with waiting time. Since, the free-energy landscape exhibits many metastable states, the system may explore all these possible states before finding the minimum energy configuration. In this process, it may end up forming gels, glasses and/or mixed states depending on the concentration of solid particles, the interaction strength and the waiting duration. We refer to particles being in a glassy out of equilibrium state, when a solid phase with a yield strength has formed in which the particles’ motions are constrained^6,11–13^. Recent observations on Laponite systems show two distinct glass states at two characteristic aging times, first one, dominated by long-range screened Coulombic repulsion (Wigner glass) and a second one, stabilized by orientational attractions (disconnected House of Cards glass)^12^. We refer to the particles to be in gelled state, when the self-assembly of the particles develop a yield stress through pair-wise attractive interactions with neighbours although driven rearrangements are still possible. The real distinction in the definition between glassy and gels states of particles self-assembled through attractive interactions is still under debate.

Recent studies on Na-Montmorillonite (MMT) and Laponite showed that these clays can form equilibrium gels under certain conditions, where the phase is completely reversible on small changes in parameters such as interaction potential^14,15^ similar to patchy colloids^16,17^. This behavior is attributed to the “patchy nature” of these materials^18^. Na-Montmorillonite is a natural bentonite and Laponite^®^ is made synthetically with a reasonable control on the polydispersity (Fig. S1). Use of mixed clays of Laponite^®^ and MMT for tuning the rheology of hydrogel nanocomposites, and for designing enzyme-free electrodes is rising^8,19^. In this study, we use binary aqueous dispersions of nanoclays to form the gel, composed of synthetic hectorite (Laponite^®^, platelet diameter of 25 nm, and rim thickness of 1 nm) and natural montmorillonite (Na-MMT, average diameter of 250 nm, and rim thickness of 1 nm), which are both clay minerals with different properties shown in Table S1 (SI). In this study, we have used fractionated Montmorillonite referred as FMMT hereafter. Recently, we have reported the evolution of various phases namely gels, repulsive glasses and locally ordered glasses with interesting aging dynamics and intrinsic anisotropic ordering^20–22^ in mixed clay systems similar to that of Laponite^®^ (L)^23–25^. Yogesh *et al*. have extensively studied the physicochemical effects in aging Laponite suspensions, prediction of rheological behavior of soft glassy materials and thermally activated asymmetric recovery in Laponite dispersions^26–29^. Avalanche behavior, viscosity bifurcation, thixotropy, shear effects jamming and flows in yield stress fluids of clays is well studied^30–32^. Lekkerkerker *et al*. explored the phase behavior of mixed hard platelets where they observed multiphase coexistence of isotropic–isotropic–nematic, isotropic–nematic and isotropic–nematic–nematic phases in mixed suspensions of large and small hard platelets^33^. These reports indicate that mixed clay dispersions, of large and small platelets, do give rise to several condensed matter phases, out of equilibrium states namely gels and glasses, and that most of these states continue to remain rich in dynamics often caused by competing interactions^34^. Our previous studies showed age dependent evolution of viscoelastic properties in nanoclay dispersions probed by rheology and light scattering, but no real space studies were performed^20–23^.

Although it is generally accepted that colloidal clay suspensions can form gels, the local gel structures are not fully investigated *in-situ*, nor is the mechanism that holds these particles together is fully understood or agreed upon. Herein, we explore the gelation mechanism and evolution of microstructure systematically in real time, and map the aging dynamics in clay (L and FMMT) dispersions prepared using various mixing ratios. The slow dynamics was captured through laser light scattering, rheology measurements, and the *in-situ* evolution of gel structure was captured using confocal microscopy. The final micro-structure was also investigated at higher resolution in real space through scanning electron microscopy (SEM). All the gel samples exhibited slow dynamics driven aging, and spontaneously approached their arrested states. Dilute samples of mixed clay dispersions did undergo phase separation into colloid-rich and colloid-poor regions similar to single component systems. No phase separation was observed in the gel samples even after months. To the best of our knowledge, there are no reports on the study of bicontinuous gelation of mixed clay systems in real space.

## Results

### Visual Observation

**Figure 1:**
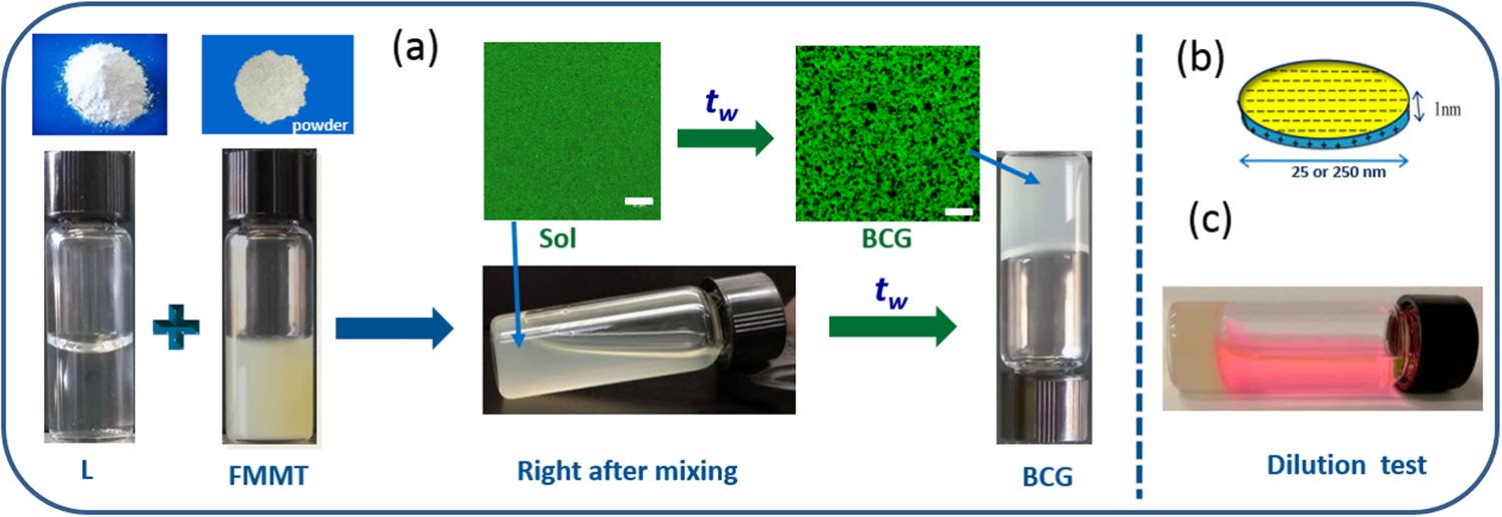
Gelation process: (**a**) Samples in the powder form, dispersions of individual systems and sol state of mixed system that eventually form bicontinuous gels (BCG). (**b**) Structure and charge distribution of clay particle, (**c**) Dilution test was performed on samples after reaching a bicontinuous gel state, doesn’t show melting upon addition of water indicates the presence of attractive interactions. A fluorescent dye was added to water to distinguish gel from solvent.

As shown in Fig. 1, after mixing the dispersions of the two clays (prepared using different mixing ratios), it was found to be in the sol form, but eventually with waiting time the structure between L and FMMT coarsened in all directions (formation of inter-platelet complexes driven by different interactions, namely FMMT-L-FMMT or L-FMMT-L) giving rise to a 3D-interconnected network. Gelation of the mixed clay system was studied at low salt concentration of 5 × 10^−4^ M NaCl and at pH = 8.5. Solution state properties were probed in our previous study^22^. The complexes showed improved mechanical and thermal properties compared to their individual systems. As the sample aged the physical properties kept evolving until a phase was achieved that did not change at the time scale of the experiment.

In this study, all concentrations are expressed in terms of volume fractions *ϕ* of clay particles dispersed in water and a mixing ratio *r* is defined as the ratio between the volume fractions of two clays. Volume fraction of L (*ϕ*_L_) is calculated from the weight percent *w* of the particles using eq. 1,

1 $${\varphi }_{L}=\frac{{w}_{L}/{\rho }_{L}}{{w}_{L}/{\rho }_{L}+{w}_{FMMT}/{\rho }_{FMMT}+{w}_{wat}/{\rho }_{wat}}$$

An equivalent expression is obtained for FMMT to calculate *ϕ*_FMMT_. The ratio between the volume fractions of the components is defined as *r* = *ϕ*_FMMT_/*ϕ*_L_. Samples made from L and FMMT did not show any phase separation or sedimentation before mixing in different proportions to form the bicontinuous gel.

### Phase diagram and Viscoelastic studies

Due to their smallness, nanoclay platelets have a relatively large diffusion coefficient making the dynamics faster than for systems built from larger particles. A typical phase diagram of mixed system is shown in Fig. 1a, where we have identified different phases based on the rheological and real-space experiments, dilution tests and the visual observation. The phase diagram was achieved over a span of a year and half. For low concentration samples, we have evaluated the final phase of the mixed particle dispersions by two experiments: (a) measurement of the scattered intensity as a function of time where the intensity decreased with the waiting time *t*_*w*_ and (b) visual observation of distinct sedimentation of the particles collected at the bottom of the cell. Such a phase separation was not observed at concentrations where the gels or glass were formed.

The distinction between the gel and glass is customarily identified by performing the dilution tests. Upon addition of the same solvent to the gel or glass in the vial, if the sample does not melt or get diluted, attractions are dominant in that system which we considered as gel and if it melts the sample, repulsive interactions are dominant and we consider it as glass. Dilution experiments helped to distinguish a Wigner glass from attractive glass. However, it needs further detailed investigations to establish these states using scattering experiments such as small angle x-ray scattering. Real space images using SEM and confocal, showed highly porous network structures in the gel state. Rheological measurements showed the solid nature of the gels and glasses from the frequency sweep response. Waiting time *t*_*w*_ is defined as the time elapsed between the sample preparation and measurement. Viscosity growth of the dispersions as function of waiting time is shown in Fig. S2. The viscosity growth profile of the gelling co-sols was intermediate between the two single component dispersions.

The viscosity was found to grow as a power-law for the dispersions at all mixing ratios given by $\eta \, \sim \,{t}_{w}^{\alpha }$. Complex viscosity and elastic modulus also followed the power-law behavior (Figs S2 and S3). Elastic modulus and viscosity exhibited inverse dependence on *r*. The viscosity of the dispersions for small aspect ratio particles was always higher than the particles with high aspect ratio for the same volume fraction. Viscosity of the dispersion was affected by the particle size, shape and temperature. In the mixed systems the effect of excluded volume was discussed in our previous work^20,21^. The scattering intensity, from the light scattering experiments, showed a linear relationship with the concentration of clays. Thus, it was argued, from the theory of scattering that the platelets of Laponite, FMMT and the mixed system are associating in an excluded volume environment^21^. As the time progressed the particles changed into larger and larger clusters and the particles lost their individual characteristics and formed a percolating network structure, or got trapped in the locally crowded environment such as in a glass state. It was also found that the addition of L to the FMMT particles, even at a volume fraction of 0.0009 induced gelation.

**Figure 2:**
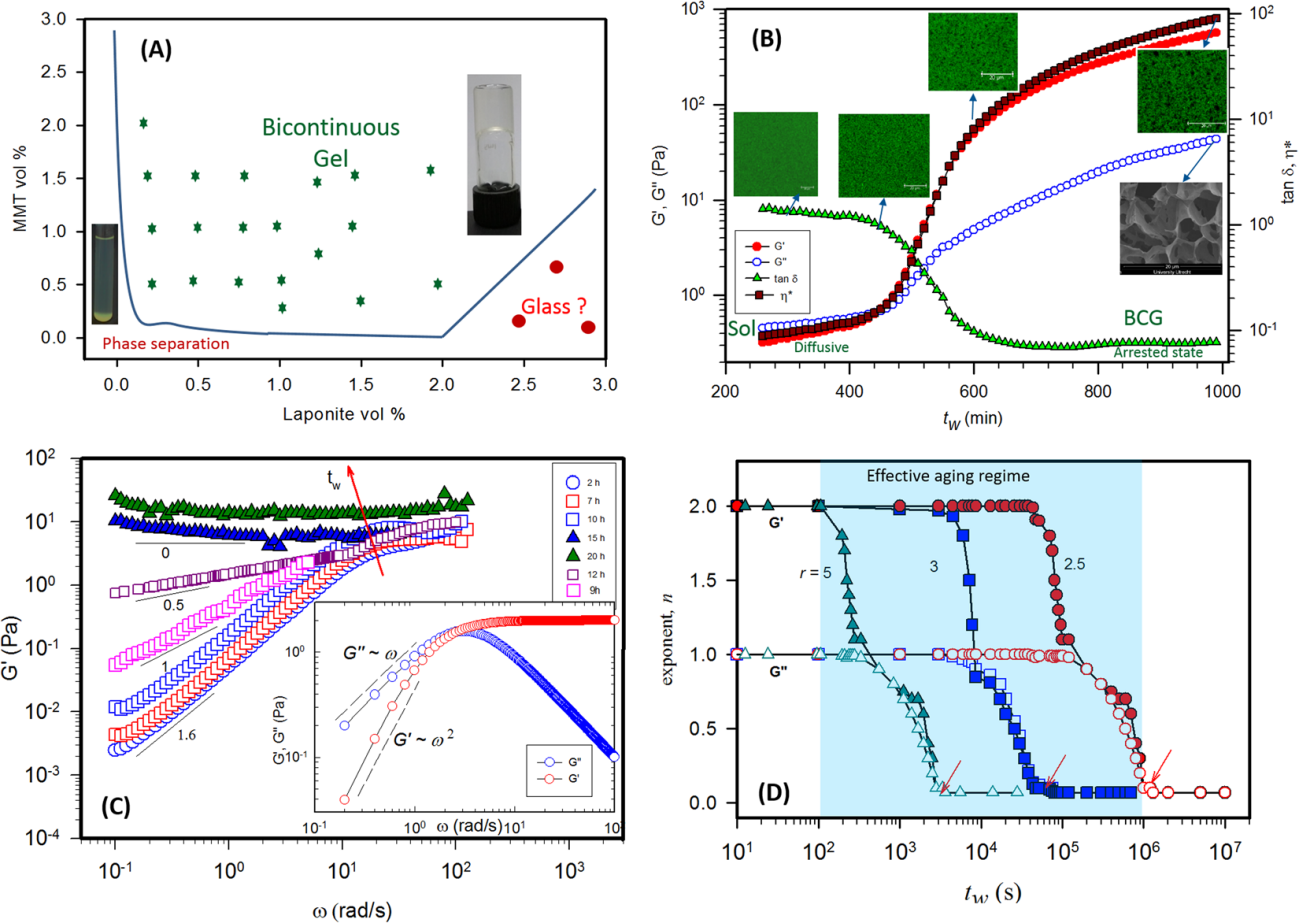
Aging dynamics of the bicontinuous gels: (**A**) Phase diagram of binary clay system, (**B**) Evolution of the elastic and loss modulii, viscosity and loss tangent as function of waiting time for *r* = 10 sample. Evolution of network structure *in-situ* was probed at different aging times using confocal microscopy and the SEM image of the gel is shown at long waiting time. (**C**) The storage modulus reached a plateau at long waiting times. Viscous modulii are not shown to retain clarity of the plot. Inset shows Maxwellian behaviour for the freshly prepared samples. (**D**) Evolution of the exponent *n* (slope of *G*′ *and G″ vs ω* curves at low frequency) shown as function of waiting time for various mixing ratios.

We applied the standard protocol of shear rejuvenation to the clay systems^35^. The sample was first loaded onto the Rheometer, using solvent trap for conducting long time aging experiments. The sample was first shear melted at a shear rate of 800 s^−1^ prior to the measurements. Isothermal frequency sweep experiments were performed at different waiting times the data for which is shown in Fig. 2C. It was found that the samples showed Maxwellian viscoelastic behaviour in the initial aging period. According to the Maxwell model, the in-phase modulus *G*′ (storage modulus) and out of phase modulus *G″* (loss modulus) as function of frequency are given by^36^

2 $$G\text{'}=\frac{{G}_{0}{\omega }^{2}{\tau }^{2}}{1+{\omega }^{2}{\tau }^{2}},\,G\text{'}\text{'}=\frac{{G}_{0}\omega \tau }{1+{\omega }^{2}{\tau }^{2}}$$

where *G*_0_ is the plateau elastic modulus, *τ* is the mean viscous relaxation time, and *ω* is the angular frequency. In the limiting case of *ω→* 0, the relations will become *G*′ ~ *ω*^2^ and *G″* ~ *ω* and the exponents of the moduli are considered signatures of the Maxwellian model. In our samples, deviation from Maxwell behaviour was observed with increase of waiting time which was probed systematically. The exponents started decreasing with waiting time from 2 and 1 for *G*′ and *G″* respectively in a structured manner, and reached a plateau at long *t*_*w*_ indicating freezing of the relaxations and the accompanied dynamic arrest of the system. This is clearly illustrated in Fig. 2D. An interesting evolution of slopes was found in the aging systems. When the system was fully aged it became solid and the frequency dependency vanished, a conclusion that was universally noticed for all the sample compositions probed in this work. An interesting evolution of inter-connected structure was observed from confocal microscopy and SEM measurements (Fig. 2B). During the initial aging times, there were no specific patterns in the confocal images and the solution was homogeneous but as the waiting time progressed few bright spots started to appear. Interestingly the appearance of such network structure started close to the cross-over point, where the viscosity divergence or the *G*′ build up occurred. Age dependent evolution of microstructure is systematically studied in the later sections.

This data indicates that the samples although were not chemical gels in the sense that no chemical bonds had formed between the particles, the interparticle interactions were so strong that the relaxation time of the system occurred over a time scale that was beyond the available experimental window. The power-law scaling of the modulus with frequency suggested self-similar relaxation which is expected of hierarchical gels^37^. The cross-over time provides an approximate measure of the gelation time, because the frequency dependence of G′ and G′′ are scaled independently by the same factor, *cos(n*_*c*_*π/2)* = *sin(n*_*c*_*π/2)* = 1/√ 2 for *n*_*c*_ = 0.5. It is clear from the above discussion that the system which was initially in a liquid state transits through a critical gel state and became solid-like (arrested) beyond the gelation time. However, keeping a physical gel at the critical gel state is not possible, as it will keep on evolving to minimize its free-energy. It continues to evolve until the elastic modulus becomes independent of frequency which means it gains the properties of that of a Hookean solid.

For a gelation process driven by strong attractive interactions (*E*/*k*_*B*_*T* >> 1) (*E* is the depth of the attractive potential) the initial sol state transforms into a gel at a well-defined gelation time *t*_*gel*_. At *t*_*gel*_ the system exhibits a behaviour specific to percolation such as a power-law frequency dependence of complex modulus which reflects the percolation transition. The critical gel state is the state where the formation of space-spanning network takes place. According to Winter-Chambon proposition^38^, at the critical point, dynamic viscoelastic modulii (*G*′, and *G″*) exhibit identical frequency dependence given by

3 $$G\text{'}(\omega )=G^{\prime\prime} (\omega )\,\cot (n\pi /2)=[\pi S/2\Gamma (n)\,\sin (n\pi /2)]\,{\omega }^{n}$$

where *Γ(n)* is an Euler gamma function of power-law exponent *n* (0 < *n* < 1) and *S* is the gel strength. At the critical gel state tan *δ* = tan (*nπ*/2). Therefore a critical state is identified as the point where iso-frequency tan *δ* curves meet. According to Winter, for a stoichiometrically balanced end-linking network *n* = 0.5, while for crosslinked stiff gel *n* decreases and for soft gel *n* increases. There have been numerous systems that validate the Winter-Chambon criterion of the critical gel state, namely crosslinked polymers, colloidal gels, and recently Laponite clay gels^39,40^. The critical gel state is known to have fractal structure and there is a relationship obtained by Muthukumar^39^ between the power-law exponent and fractal dimension *d*_*f*_ by accounting for excluded volume interaction and for a *d*-dimensional system, it is given by *d*_*f*_ = (*d* + 2)(2*n* − *d*)/2(*n* − *d*). The fractal dimension *d*_*f*_ varied between 2.0 and 2.15 depending on the value of *r*. These values are not too different from the Laponite systems studied by Joshi *et al*.^40^.

### Dynamic Light Scattering

**Figure 3:**
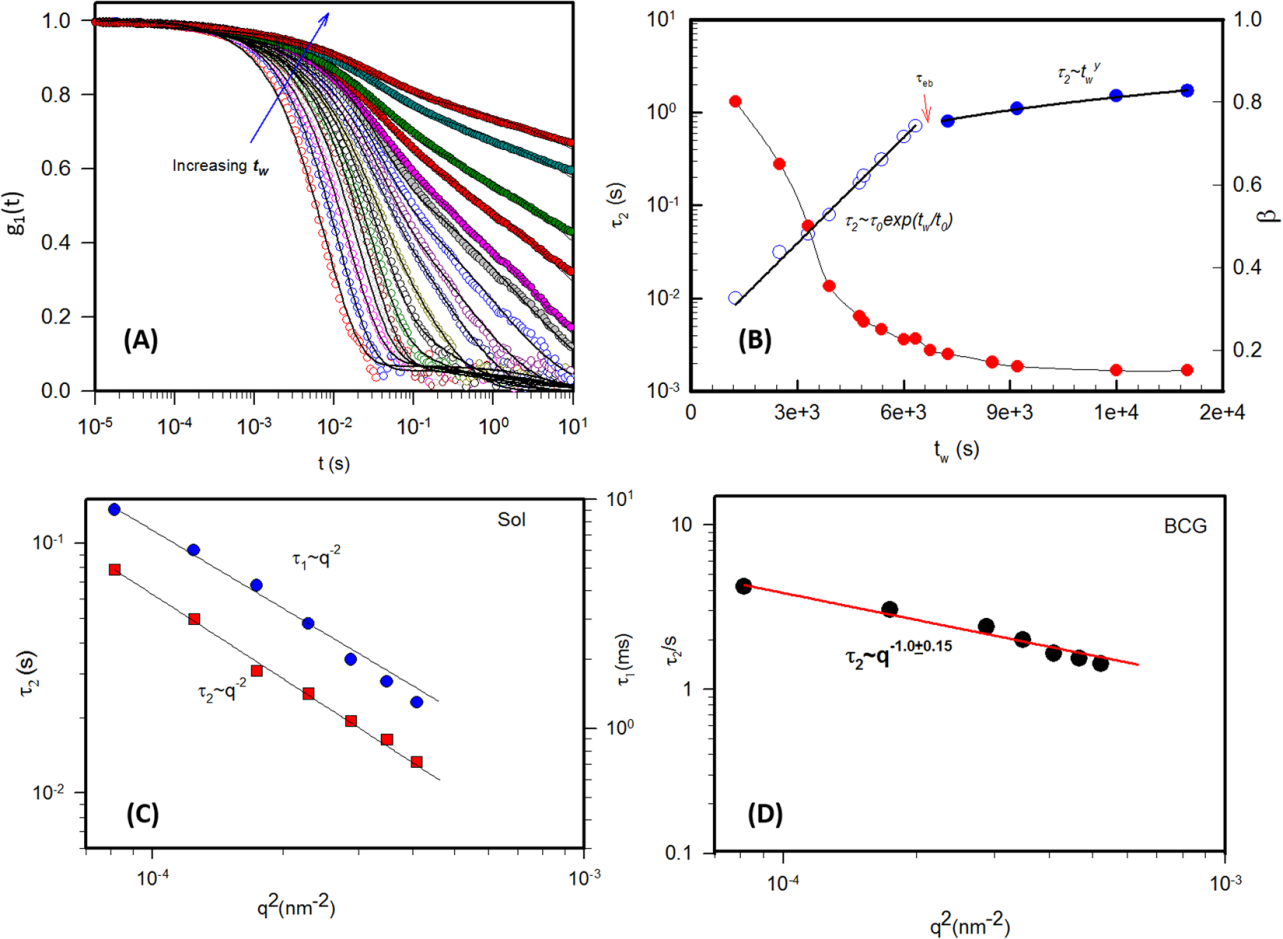
Study of aging behavior of BCGs from DLS: (**A**) Evolution of the dynamic structure factor as function of waiting time *t*_*w*_ for *r* = 5 sample. The arrow indicates the direction of increment in waiting time. (**B**) Slow mode relaxation time and the stretch exponent *β* as function of *t*_*w*_. Note the two regions are differentiated by an ergodicity breaking time *τ*_*eb*_. Fast and slow relaxation times (*τ*_1_ and *τ*_2_ respectively) vary inversely with the square of wave vector in sol (**C**) and gel states (**D**). Size of the symbol is the error in the measurement.

Aging dynamics of dispersions were best probed using dynamic light scattering techniques by evaluating the dynamic structure factor with waiting time (Fig. 3A). In arrested, non-ergodic, systems, determination of the dynamic structure factor from the recorded intensity correlation data poses a serious problem because of non-applicability of the Siegert relation. Several different methods have been reported to deal with the non-ergodic systems using DLS^41–47^. In this study, this was handled by taking out the non-ergodicity component from the measured data using the modified heterodyne method presented by Sellen and others^48–50^. In this method a position of the cuvette is selected such that the time averaged scattered intensity <*I*>_*T*_ is equal to the ensemble averaged intensity <*I*>_*E*_. Here <*I*>_*E*_ was obtained from the sample rotation data. Deviations between the averages of less than 5% will not influence the results largely. Here, the degree of spectral broadening, *X* could be calculated from the intercept (*σ*^2^) of the obtained correlation function and *β*′ from rotation,

4 $$X=1-{(1-{(\frac{{\rm{\sigma }}}{\beta \text{'}})}^{2})}^{1/2}$$

The quantity 1 − X gives a measure of the degree of ‘frozen-in’ components which is responsible for the non-ergodicity of the system. The dynamic structure factor *g*_1_(q, t), now describing only the dynamic parts after neglecting the ‘frozen-in’ parts was calculated as

5 $${g}_{1}({\rm{q}},\,{\rm{t}})=\frac{{\rm{X}}-1}{X}+{[{(\frac{{\rm{X}}-1}{X})}^{2}+\frac{{g}_{2}({\rm{q}},{\rm{t}})-1}{{(\beta \text{'}X)}^{2}}]}^{1/2}$$

where *g*_1_(q, t) is the time–averaged correlation function obtained at a position where approximately <*I*>_*T*_ = <*I*>_*E*_.

In general the dynamics structure factor *g*_1_(*q*, *t*) in clay systems is described by a two-step relaxation given by^12,21^

6 $${g}_{1}(q,\,t)=a\,exp[-(\frac{t}{{\tau }_{1}})]+(1-a)\,exp[-{(\frac{t}{{\tau }_{2}})}^{\beta }]$$

where *a* and (1 − *a*) are the weights of the two relaxation modes defined by relaxation times *τ*_*1*_ and *τ*_2_, which represent the fast and the slow mode relaxations respectively, and *β* is the stretching parameter. The fast mode relaxation time *τ*_1_ is related to the inverse of the short-time diffusion coefficient *D*_*s*_ as *τ*_1_ = *1/D*_*s*_ *q*^2^. The data shown in Fig. 3A could not either be fitted to a single exponential or with a single stretch exponential. The best least-squares fits were obtained when the data was fitted to single and stretched exponential as given by eq. 6.

The waiting time dependent evolution of the dynamic structure factor for gel samples is shown in Fig. 3A. For times less than the ergodicity breaking time, the dynamic structure factor decays to zero, the fast and slow relaxation times vary inversely with the square of wave vector (*τ*_1_*, τ*_2_ *~ q*^−2^), shown in Fig. 3C, a feature reminiscent of diffusive motion. Beyond this ergodicity breaking time the slow relaxation time varied inversely with the wave vector (*τ*_2_ *~ q*^−1^) as shown in Fig. 3D which indicated anomalous diffusion. The characteristic fast mode relaxation dynamics corresponds to interaction between a particle and cage of its nearest neighbours, it remained invariant during the aging period that we studied. The characteristic slow mode relaxation time was found to grow exponentially with waiting time *t*_*w*_. The temporal evolution of the slow mode relaxation time τ_2_ is presented in Fig. 3B for *t*_*w*_ *<* *τ*_*eb*_ which could be described by *τ*_2_ ~ *τ*_0_exp(*t*_*w*_/*t*_0_). The temporal evolution of slow mode relaxation time beyond *τ*_*eb*_ (*t*_*w*_ > *τ*_*eb*_), followed a power-law linear growth given by ${\tau }_{2}\, \sim \,{t}_{w\,}^{y}$, where *y* varies from 0.97 to 1.1 depending on the concentration of the sample, though the range of waiting time is limited in the second regime.

The two approaches by heterodyne method and Pusey and van Megen method look similar at first sight but they differ significantly by the way the normalization of intensity correlation is done and they require different evolution procedures. Almost identical results for the relaxation times were obtained in both cases which was demonstrated by Rodd *et al*. for Xanthan gels^49,50^. Data from these two approaches showed that they are in good agreement within the experimental error. Khavari *et al*. also found that both the modified heterodyne and the brute force, rigorous Pusey-van Megen methods gave close to the same values of the relaxation times^50^.

### ***In-situ*** gelation kinetics by confocal laser scanning fluorescence microscopy

**Figure 4:**
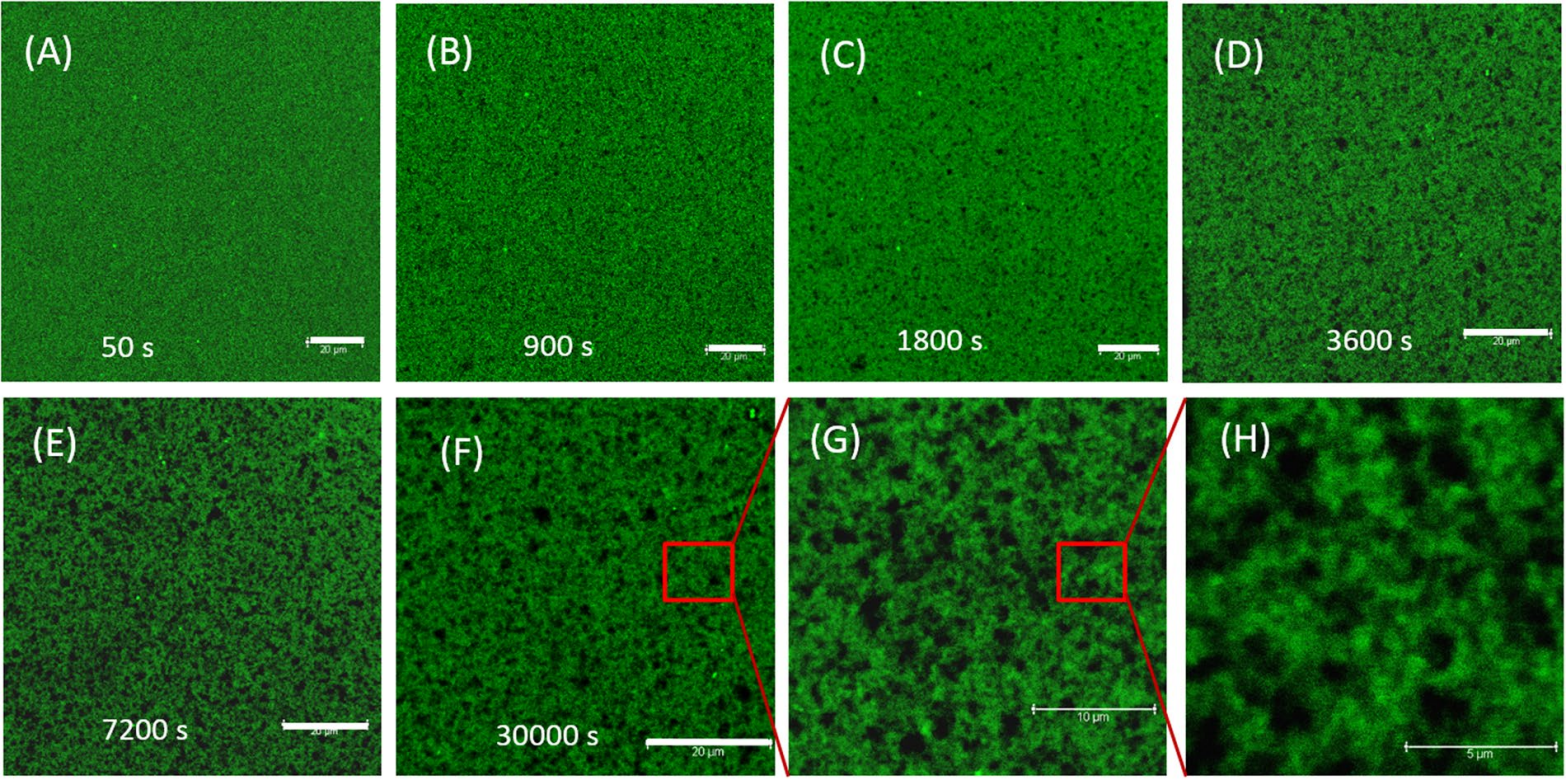
Temporal evolution of the microstructure during gelation: Confocal laser scanning fluorescence microscope images of the gelation process of *r* = 5 sample. Scare bar is 20 μm.

In order to explore the self-assembly of the platelets leading to bicontinuous gelation from the homogeneous sol phase, fluorescence confocal imaging experiments were performed as function of waiting time. Rhodamine 6 G was used in fluorescence labeling of Laponite which exhibited good fluorescence in aqueous medium. Our studies indicated that the gelation process was not influenced by the presence of Rhodamine 6 G. We performed rheological measurements on the fluorescently labelled gels and compared it with non labelled samples to see if there was any significant change in their viscoelastic properties, and we found no difference. The labeled Laponite particles were mixed with FMMT to produce different samples of desired mixing ratios. The gelation process was visualized and recorded over different scanning areas at a resolution of 512 × 512 pixels^2^. For waiting times below 500 s high scanning speeds, and for waiting times above 500 s low scanning speeds were employed. No patterns were observed during the initial aging times, but as the waiting time progressed few bright spots started to appear of which the number increased with the waiting time, and gradually a connected network-type structure was observed as shown in Fig. 4.

**Figure 5:**
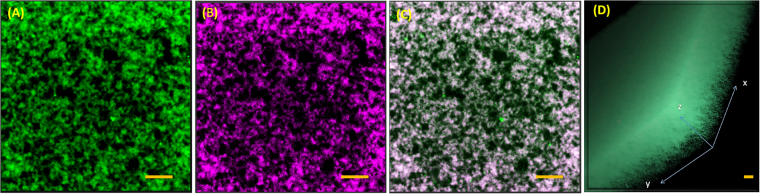
Structure of BCGs: Gel structure from fluorescent L (**A**), FMMT (**B**) and overlapped structure (**C**) from A and B for mixing ratio *r* = 4.5. (**D**) 3D structure of *r* = 5 obtained by combining 2D slices of confocal images which shows the porous nature of the network present in the BCG. Scare bar is 20 μm.

**Figure 6:**
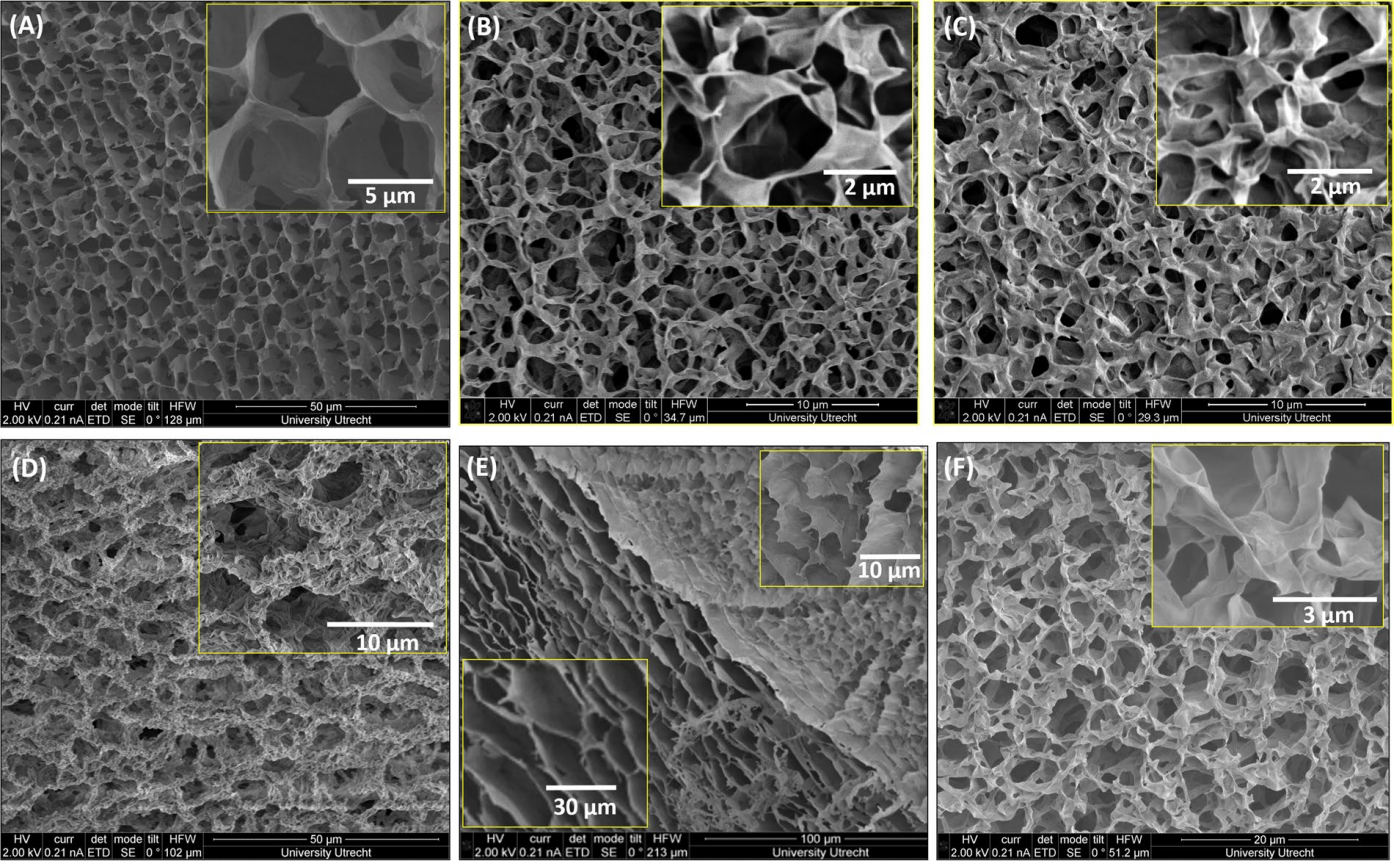
SEM images of the BCGs for different mixing ratios: (**A**) 10, (**B**) 5, (**C**) 4, (**D**) 3, (**E**) 2.5, and (**F**) FMMT gels (*φ*_*FMMT*_ = 0.0011). The insets show the images at high-magnification.

**Figure 7:**
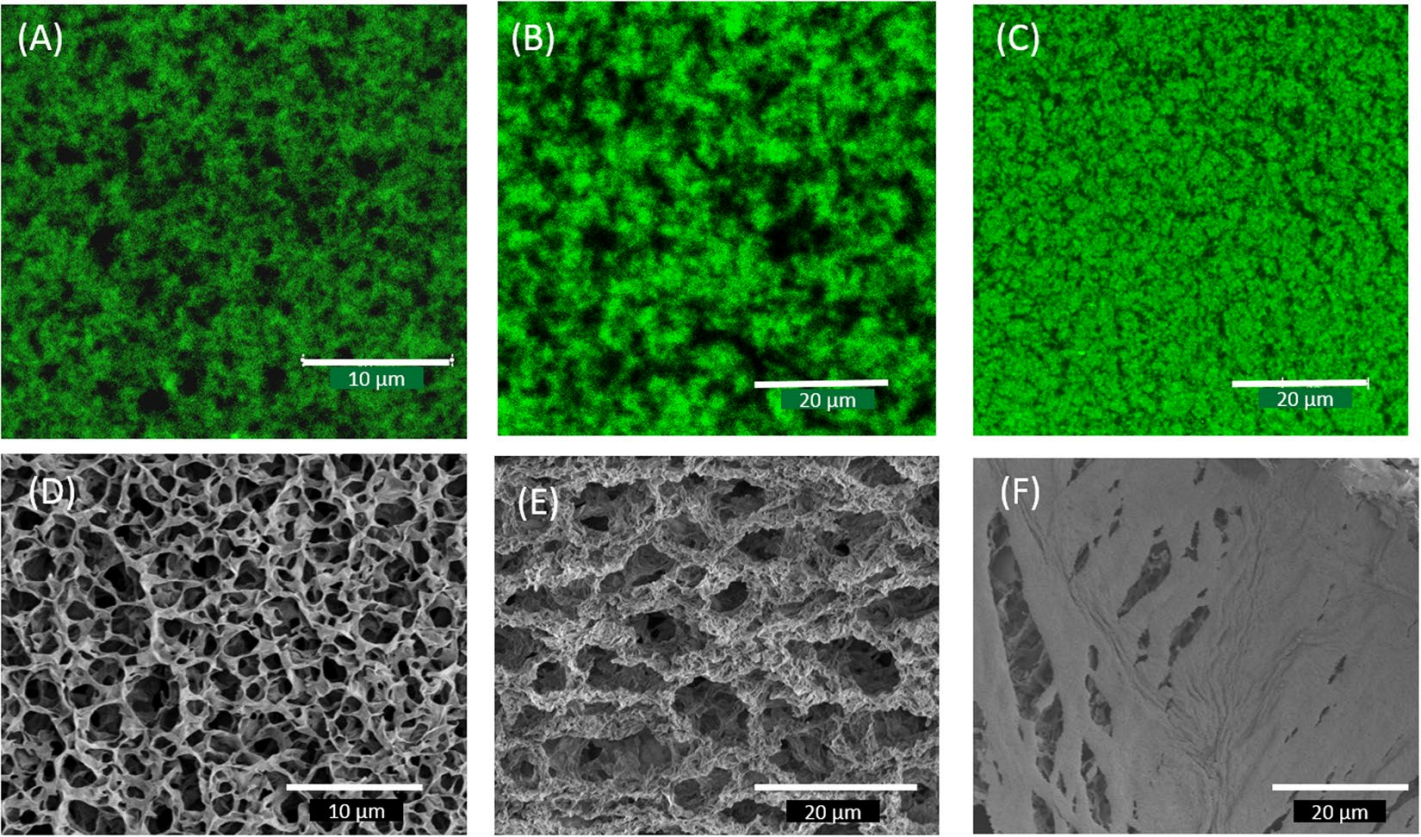
Comparison of microstructure of bicontinuous gels: Confocal images (**A**–**C**) and SEM images (**D**–**F**) for samples at *r* = 5 (**A**,**D**), 3 (**B**,**E**) and 2.5 (**C**,**F**) mixing ratios.

Interconnected network formation was clearly seen for the range of samples studied here. Thus the 3D-network formation continuously evolved from the initially homogeneous particle dispersion. Therefore, it was an interconnected network made of two interacting platelets, and the evolution of three dimensional microporous architecture that is clearly seen in the z-stack of the gels (Movie S1, Supplementary Information), and in the 3D reconstruction of 2D gel slices of confocal images (Fig. 5D). This is similar to gels found in Laponite systems, where gelation occurs through specific interactions (T-bonds) without formation of clusters^14^. In our experiments, the number of pores continuously increased with waiting time and the evolution almost stopped beyond gelation time, but the strength of the network continued to evolve due to slow aging even after the gelation point had reached. The gelation process at other mixing ratios is shown in Fig. S4 and the results are compared with the SEM measurements (Figs 6 and 7).

The gelation process can be different for samples prepared with different mixing ratios. During the initial period bigger and smaller platelets aggregated to form a network. In order to find the position of FMMT particle inside the network, FMMT particles are fluorescently labelled with Rhodamine B. Figure 5 shows the fluorescent image of BCG formed from fluorescently labeled L and FMMT particles. It shows the FMMT particles are clearly participated in the network formation. It may be assumed that the FMMT particles forms network of their own and the small L particles come close to the preformed network of bigger particles. However, this assumption may not be true always. There is a distinct possibility of exchange of dye molecules between different platelets, which needs to be explored further. The gelation time was found to depend on the concentration of the smaller particles for a given number of larger particles. The domains with colloid poor regions started to grow bigger and reached a state where the evolution of the gel was very slow. Similar observations were made from the rheology data, where the growth of the storage modulus was rapid during the effective aging period *(t*_*w*_ > *τ*_*eb*_). These observations on gelation process using confocal microscopy are consistent with many hydrogel systems^51–53^.

### Microscopic structure of the Bicontinuous Gels using scanning electron microscopy (SEM)

The microstructure of the bicontinuous gels was explored at higher resolution using scanning electron microscopy (SEM) on freeze-dried samples, to prevent drying forces to alter the gels formed. In order to explore the structure systematically and compare with the confocal results, the ratio of the volume fractions are kept the same. The SEM images show that the freeze-dried gels had porous structures. Increasing the magnification shows the gel structure had a foam-like structure with big pores at *r* = 10 (Fig. 6A). In this case the network may be formed by FMMT platelets surrounded by L particles with thin branches. At *r* = 5, higher number of FMMT particles were found to form the network with thick branches with increased number of pores (Fig. 6B). Even at low values of *r*, number of pores increased and the average pore size was found to decrease. One could conjecture that these structures were made of overlapped coins (OC) or house of cards configuration.

A closer look at the average pore size and the thickness of the branches gives useful information about the interactions prevailing in the network. The average pore size decreased with the concentration of the small particles and it increased drastically for *r* = 3 (Fig. 6D), and, say *r* = 2.5 or less, the system was found to phase separate and form networks independently from the individual components (Fig. 6E). In Fig. 6E, at higher magnification, the structure appears smooth, with some traces of submicron building blocks located along the edges. Two kinds of network structures are present in such a system as shown in Fig. 6E. SEM micrograph of a FMMT gel of *φ*_*FMMT*_ = 0.011 is shown in Fig. 6F, which indicates the OC configuration is mainly responsible for gel formation with bigger pores as was predicted by Samim *et al*.^54^. The bicontinuous gel system showed honeycomb-like structure mainly at high *r*, but it is not prominent in all cases. Thus the revealed structure resembles a honeycomb, which is most likely an artifact created by ice crystallization during plunge freezing. Number of pores per unit volume increased as a function of *r*. Similar trend was noticed from confocal imaging which is shown in Table 1. Such microporous structures have been observed in Montmorillonite gels at higher concentrations, and in graphene films^54,55^. The microstructure of the gels obtained from CSLM and SEM are compared in Fig. 7. Both images show similar behavior of formation of colloid-poor and colloid-rich regions, the presence of pores and the similarity in their microstructures. For *r* = 2.5 sample, the two images (Fig. 7C and F) are qualitatively different which needs further studies. Thus the results obtained from confocal microscopy measurements are consistent with the network structure revealed in the SEM images.

**Table 1:** Pore size and the thickness of the branch listed as function of mixing ratio.

**Mixing ratio (** ***r*** **)**	10	5	4	3	2.5
Area (μm^2^) SEM Confocal	25 ± 7 29 ± 11	3 ± 0.5 3 ± 0.7	1.3 ± 0.4 1.5 ± 0.5	47 ± 17 51 ± 20	97 ± 20, 17 ± 4 —
Thickness of branches (nm)	123 ± 3.5	70 ± 17	35 ± 8	2203 ± 300	300 ± 60, 101 ± 10

### Temperature induced transition

In order to understand the nature of the interactions responsible for network formation, we examined the effect of temperature on these gels. This was because it is known that a change in temperature changes the inter particle interactions. Prior to heating experiments, sample vial containing the bicontinuous gel was placed in a beaker containing deionized water of same ionic strength. The temperature of the system was increased at a rate of 2 °C/min to determine the precise point where the gel started to flow. The gel did not flow or got diluted at lower temperatures. The transition or yield-point temperature *T*_*c*_ is defined as a temperature where the gel shows signs of deformation or flow into water.

**Figure 8:**
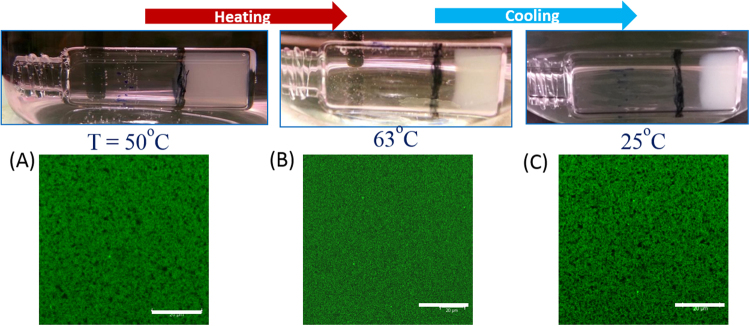
Heating experiments on BCGs: Determination of *T*_*c*_ (63 °C) for *r* = 3 gel, heating and cooling cycles are shown. Corresponding confocal images at specified temperatures are shown in the bottom panel. Scale bar is 20 μm.

No sign of deformation or melting was seen for temperatures below *T*_*c*_ (Fig. 8). At *T*_*c*_ the gels started to deform and flow, but the gel state was recovered as it was cooled to room temperature (20 °C). The *T*_*c*_ also significantly varied with the age of the gel and the mixing ratio. At higher aging times, the gels showed higher *T*_*c*_ values. The gels with *r* ≥ 3 showed a clear transition at temperature *T*_*c*_. The *T*_*c*_ of the BCGs was always higher than the gels formed of their individual clays (Table S1). Confocal images show the fractionation of the network into smaller clusters at *T*_*c*_ that were found to re-assemble into a gel structure when cooled to room temperature.

Gels made of clays are physical in nature and yet, they exhibit strong viscoelastic behaviour similar to chemical gels. Theses gels were found to age irreversibly with waiting time. As the gel was heated the organization of the particles got randomly redistributed. At higher temperatures, domains in the gel got disrupted due to the heat, thereby allowing the particles to get out of the network and the phase becomes a homogeneous sol. In order to understand the transition, we performed rheological experiments to determine *T*_*c*_ (Fig. S5). Stiffening of gels was observed during the temperature sweep experiments, similar to some hydrogels. Around *T*_*c*_ the growth of elastic modulus was very slow and also the loss modulus and loss tangent showed a clear change. Our previous studies showed that there existed a transition point, until which the ordered parameter showed an increment and remained constant beyond that.

## Discussion

We present the results for the case of mixtures of nanoclays of different aspect ratios that produced bicontinuous gels with unique attributes at low volume fractions of nanoclays at low ionic strength. Small platelets of L help the bigger particles of FMMT to bind together through directed electrostatic attractions and the structure grows with aging, coarsens in all directions giving rise to a bicontinuous gel network structure that imparts increasing viscosity to the gelling system and elastic modulus. Viscosity of the mixed dispersions had intermediate values between the individual suspensions of L and FMMT. As the concentration of the particles increased, the anisotropic interactions between the particles facilitates the formation of either T-bonds or band structure which has been discussed in previous studies^13,54^; as a result the viscosity and elastic strength of the system increases yielding a dynamically arrested phase which behaved like a Hookean solid.

Interestingly, the aging dynamics in mixed clay dispersions and gels was found to be qualitatively similar to their single component behaviour, and more than one relaxation time was noticed. During the initial stage of aging, the relaxation time was found to grow rapidly (exponentially) followed by a linear growth. A similar picture was evident from the rheological measurements, where it was found that during initial aging the system behaved as a Maxwell fluid, but as time progressed it deviated from Maxwellian behaviour and crossed over to an arrested phase indicative of the presence of a critical gel state. Beyond the ergodicity breaking time or the cross-over time the solid nature was apparent. Thus, the bicontinuous gel was made of platelets of different aspect ratios and charge densities were bound by anisotropic interactions. An interesting feature is obtained from all the complimentary techniques used in this study. As shown in Figs 2 and 3, at *t* < *t*_gel_ or *τ*_*eb*_, the system showed a sol state behavior with diffusive motion of the clay particles; at *t* > *t*_gel_ or *τ*_*eb*_, the system entered into a non-ergodic regime and interestingly it is also a non-Newtonian fluid state. Confocal microscopy images showed a clear age dependent structure and the onset of inter-connectivity could possibly be located around the cross-over or gelation point. A more comprehensive study is needed to find a possible link to the behavior of model colloidal gels by performing a qualitative study of the linear and non-linear rheology of the bicontinuous gels.

**Figure 9:**
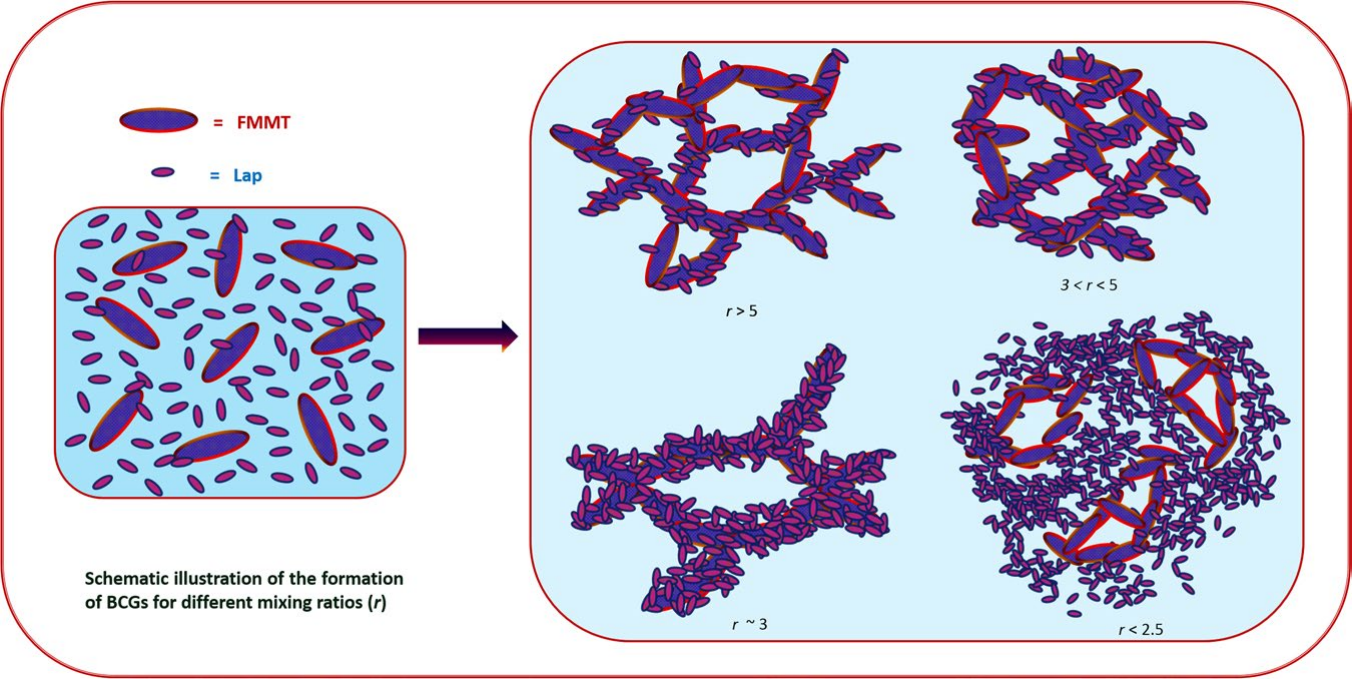
Representative depiction of the final gel structure for different values of *r* at long waiting times.

It was clearly found that in the bicontinuous gels the strength of the networks formed by the interconnected FMMT particles heavily depended on the mixing ratio, the FMMT content and the nature of the interactions prevailing between them. The FMMT particles may sit at the specific locations in the dispersion of small platelets formed network of their own. In the mixed suspensions the nature of the bigger particles is not lost and they interacted in a very specific way to build the self-assembled network of their own for any given mixing ratio, a conclusion that emerges from the Figs 5–7. The formation of network may be visualized as follows: Larger particles form the network of their own surrounded by small platelets similar to dipolar colloidal mixtures^46–48^. Amit *et al*. found through molecular dynamics simulations of equimolar binary mixtures of dipolar colloid particles, the existence of two bicontinuous gel structures: (i) the first consisting of two single-species interpenetrating networks of cross-linked chains with same dipolar strength and the second with size and low dipole moment ratio of 0.1, consisting of a network of cross-linked chains of one species sheathed by particles of the other species^56–58^, and (ii) smaller particles act as the bridges between the larger particles. Based on the observations above we proposed a representative picture of the microscopic structure of BCGs for different mixing ratios as shown in Fig. 9. Our experiments also indicated that with the addition of L particles to FMMT enhanced of network strength. We suspect that this may be due to reduction of spillover effect. Since our experiments were performed at low ionic strengths screening of the positive or negative charge of FMMT comes from the contribution of smaller particles which makes the spillover smaller thus increasing the strength of the network.

At higher values of *r*, the network was mainly formed by the larger particles and small particles helped them to increase the strength of the network by physically locating themselves at energetically favorable locations. At a mixing ratio of *r* = *φ*_*FMMT*_/*φ*_*L*_ ~ 3, a network with high thickness of the braches could be found. A similar observation was made on the dipolar colloids of different sizes which show a network of larger particles is formed initially and the smaller particles forms a sheath around the preformed network due to van der Waals interaction. At values *r* ≤ 2.5, the system could be stabilized by repulsive interactions, because the dilution experiments showed melting. In another study on mixtures of inorganic nano-sheets, the presence of clays in niobate nano-sheets also showed such a microscopic separation, which assembled into the microdomains of niobate and clay particles separately stabilized by repulsive interactions^59,60^. At low value of *r*, our system may also fall into this category of forming the micro domains of larger and smaller particles separately, which was stabilized by repulsive interactions. Our studies also probed the effect of varying the volume fractions at given values of *r* and observed similar trends in the evolution of microstructure and dynamics.

The effect of temperature on the bicontinuous gels was studied to map the interactions responsible for the network formation. Confocal micrographs show the rupture of the network as the transition temperature was approached as shown in Fig. 8. Rheological measurements showed the stiffening of the gels with the temperature. It may be due the modification of the interactions between the clay particles or the breakdown of the clusters which may form the attractive glass. Our previous studies showed that in the heating experiments there existed a transition temperature until which the anisotropic ordering in the system increased and remained constant beyond *T*_*c*_^22^. If the system shows such ordered glassy behavior, it would be melted by the addition of water as was observed in the dilution experiments. Here, it may be assumed that the networks were affine that allows the gel content to be described through, its degree of crosslinking to a first approximation. An affine network embodies the following properties: (i) the relaxed network is isotropic, (ii) molecular level deformation is same as at the macroscopic level, (iii) volume is invariant of deformation, and (iv) network entropy is the same as the sum of individual component entropies. At the microscopic scale, the gel domains will experience the same heat bath condition during the heating experiment. This will cause the local gel domains to reorganize due to acquired higher mobility of colloid particles, which in turn will tend to increase their inter-particle distance. Thus, the interactions will weaken causing the gel domains to collapse at microscopic scale. Globally, this will be observed as a melting transition or the formation of ordered glass. Hence, our study of the temperature dependence was interesting as well as educative, and hence changing the interactions between the clay particles by temperature needs to be researched in more detail. Temperature dependent transitions continue to play a significant role in soft matter systems such as clays to induce thermal transitions, and modulate their viscoelastic nature. Upon drying the bicontinuous gels, thin films were formed with varying smoothness of the surface as a function of mixing ratio (Fig. S6). We envisage that the thin films made from the bicontinuous gels may enhance the flame resistance and gas barrier properties.

## Conclusion

To conclude, we report the *in-situ* visualization of the formation of bicontinuous clay gels formed from a binary mixture of anisotropic platelets of different aspect ratio and charge density mixed at various ratios. We comprehensively studied their aging dynamics and microstructure evolution, which yielded an array of self-assembled soft matter states. Initially, mixed clay dispersions showed Maxwellian viscoelastic behaviour, which after going through the gelation transition showed considerable deviation. Dynamic structure factor captured two distinct relaxation modes namely, fast and slow modes. During the initial aging the slow mode relaxation time increased exponentially while in the later aging period it grew linearly similar to model colloidal gel and glassy systems. Microscopic studies allowed us to understand the self-assembly of the networks formed by the binary clays, where the larger and smaller platelets interact preferentially to form a network. Confocal microscopy allowed us to visualize the gelation process *in-situ*. Similar conclusions emerged from scanning electron microscopy pictures taken on these gels after the solvent was sublimated where the pore size of the gel was found to change with the mixing ratio. We also found a temperature induced transition in the mixed clay gels that signifies a change in the interparticle interactions that needs further investigation. This work is a starting point to understand the gelation mechanism of mixed clay/colloidal systems which could be extended to explore the gelation mechanism of other mixed soft matter systems. This investigation indicates this new class of clay gels can have unique and tunable physical properties that can be used for designing lithium ion batteries with low cost and high performance, or in the nuclear waste management protocols, to name a few. Bicontinuous gels could also be used to improve flame retardant properties of nanocomposites. In addition, the general understanding of self-assembly of anisotropic platelets is a topic of immense scientific interest to connect structure property relationships in systems of increasing complexity.

## Experimental Section

### Material and Methods

The nanoclays, Laponite^(R)^ RD and Cloisite-Na (or Na-MMT) were graciously gifted by BYK Additives & Instruments, Germany and used without further modification. Fractionation of MMT was done as described in our previous work^20^ since the native clay has many fractions ranging from a few nanometres to several micrometres. The selected fractionation yielded an average lateral size of 250 nm. The conductivity of the sample was brought down to 5 *µ*S cm^−1^ by repeated centrifugation. The average size of the Laponite particles was 25 nm (face diameter). Aqueous dispersions with different concentration (0.1–3.0% (w/v) of L and FMMT were prepared by dissolving L and FMMT powder separately in desired amounts in deionized water at room temperature (25 °C) and mixing them vigorously for 2 h using a magnetic stirrer. Laponite samples were filtered through 200 nm Millipore filter to remove clusters, and then the filtrate was sonicated for 5 min to homogenize the samples. Samples of mixed nanoclay dispersions were made by mixing the clay concentrations of 0.10–3.0% (w/v) of L and FMMT in different proportions to generate binary mixtures of different mixing ratios (*r* = *φ*_*FMMT*_/*φ*_*L*_, from 1 to 10). The dispersion pH was adjusted to 8.5, and the ionic strength to 5 × 10^−4^ M NaCl. The studies were done on samples by fixing *φ*_*FMMT*_ and varying *φ*_*L*_ which generated different *r* values. We also considered the effect of varying the volume fractions at a given value of *r*.

Rheological measurements were performed on a controlled stress AR-500 model Rheometer (TA Instrument, England) using small oscillatory shear in the oscillatory mode. A stainless steel plate-cone geometry (radius 25 mm, and 2°) was used during the experiments. Linear and nonlinear rheology tests were performed in flow and frequency sweep modes. It is a Standard Operating Procedure in all the rheology measurements to apply a thin layer of low viscosity silicone oil on the free surface to prevent evaporation for long aging and heating experiments. Additionally, solvent traps were used for our experiments. Measurement of viscosity was done using a Sine-wave Vibro Viscometer (model – SV10, A and D Company, Japan).

Dynamic Light Scattering (DLS) experiments were performed with a 256 channel digital correlator, (PhotoCor Instruments, USA) that was operated in the multi-tau mode (logarithmically spaced channels), the time scale spanned 8-decades, i.e. from 0.5 μs to 10 s. A 35 mW linearly polarized He:Ne laser was used as excitation source. A Glan Thomson analyzer was used in front of the photomultiplier tube to enable collection of polarized light (*I*_*VV*_). The probe length scale is defined by the inverse of the modulus of the scattering wave vector *q*, where the wave vector *q* = *(4πn/λ) sin(θ/2)*, the medium refractive index is *n*, excitation wavelength is *λ* (=632.8 nm) and *θ* is scattering angle. Further details on light scattering can be found in refs^13,61^. We performed DLS for the low concentrated clay gels in order to minimize the multiple scattering effects.

The *in-situ* fluorescent images of gelation process were obtained from a confocal laser scanning fluorescence microscope (Leica SP8 with a 100 X, NA = 1.4) using an oil immersion Leica confocal objective. Initially Laponite was mixed with 5 × 10^−5^ M Rhodamine 6 G and stirred until the dye became attached to the Laponite surface (the dye was completely adsorbed onto the Laponite particles and no free dye was left in the aqueous phase due to the low concentration of dye used)^62,63^. This dispersion was then mixed with FMMT dispersion in desired concentrations, and experimental samples were taken in glass capillary for the confocal measurements. During measurements samples were placed in the borosilicate glass capillaries (VitroCom, 0.2 × 2 × 50 mm^3^) mounted on a glass cover slide (Thermo Scientific) and sealed with UV glue (Norland Optical Adhesive 68).

Particle morphology was examined by a JOEL 2100 F transmission electron microscope (Digital TEM with image analysis system and maximum magnification of 150 000x), operating at a voltage of 200 kV. The clay dispersions were drop-cast onto a carbon coated copper grid which was air dried at room temperature (20 °C) before loading onto the microscope.

SEM was employed to study the microstructures of hydrogels in freeze-dried state on a FEI Nova Nano Lab at 2 kV. To prepare freeze-dried samples, gels were aged for 48 h, and then freeze-dried at −90 °C under a vacuum 10^−6^ Pa for 6 h to sublime the water. To observe the cross-sections, freeze-dried hydrogel samples were fractured and stuck to the sample holder. All the samples were sputter-coated with 4 nm platinum before observation. The average pore size was measured by hand using the TEM imaging platform iTEM (version 5.0, Soft Imaging System GmbH) as shown in Fig. S7 (SI).

## Electronic supplementary material

Supplementary information

Movie S1: z-stack of bicontinuous gel
