# Supplementary material for: In-situ Observation of Hierarchical Self-Assembly Driven by Bicontinuous Gelation in Mixed Nanodisc Dispersions
Source: Sci Rep. 2018 Apr 3;8:5589. doi: 10.1038/s41598-018-23814-4 (PMC5882853; doi:10.1038/s41598-018-23814-4)
Supplement: Supplementary file 1 — Supplementary information [file 41598_2018_23814_MOESM1_ESM.pdf]

Supplementary Information

In-situ Observation of Hierarchical Self-Assembly Driven by Bicontinuous Gelation in Mixed Nanodisc Dispersions

Ravi Kumar Pujala[1,2,3]*, C.T.W.M. Schneijdenberg[1], Alfons van Blaaderen[1]and H. B. Bohidar[3,4]

[1]Soft Condensed Matter group, Debye Institute for Nanomaterials Science, Utrecht University, Princetonplein 5, 3584 CC Utrecht, The Netherlands

[2]School of Physics, University of Hyderabad, Hyderabad 500046, India

[3]Polymer and Biophysics Laboratory, School of Physical Sciences, Jawaharlal Nehru University, New Delhi, India

[4]Special Centre for Nanosciences, Jawaharlal Nehru University New Delhi, India

*Corresponding author email: pujalaravikumar@gmail.com

Supplementary Movies:

Movie S1: This movie shows the z-stack of bicontinuous gel for *r* = 5, shows the porous structure with well-defined network made of two nanoclays.

Characterization of nanoplatelets

Chemical structure of Laponite $Na^{+0.7}[(Si_8\,Mg_{5.5}\,Li_{0.3})\,O_{20}\,(OH)_4]^{-0.7}$

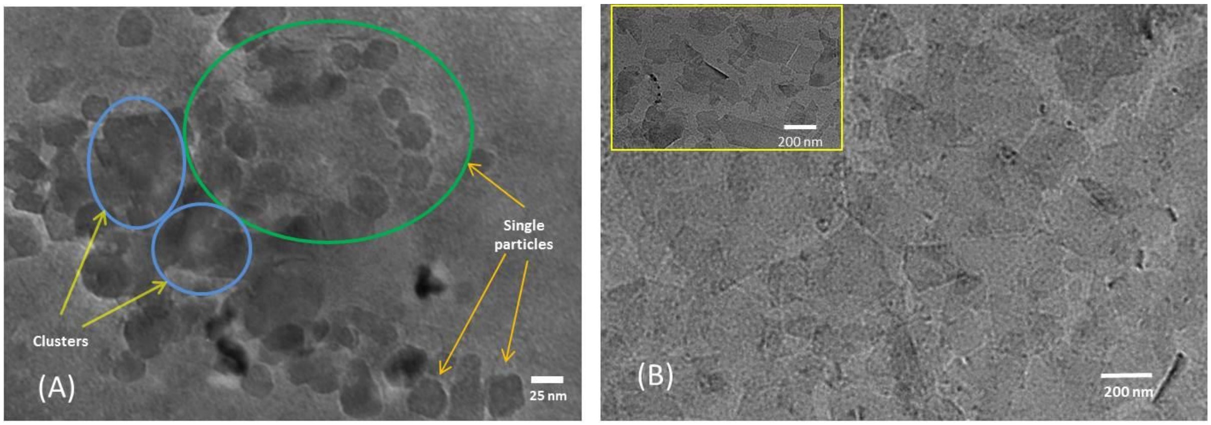

Figure S1 TEM images of Laponite (A) and Na-Montmorillonite (B). The individual particles and clusters are indicated as shown in figure. Laponite clay is monodisperse compared to Montmorillonite.

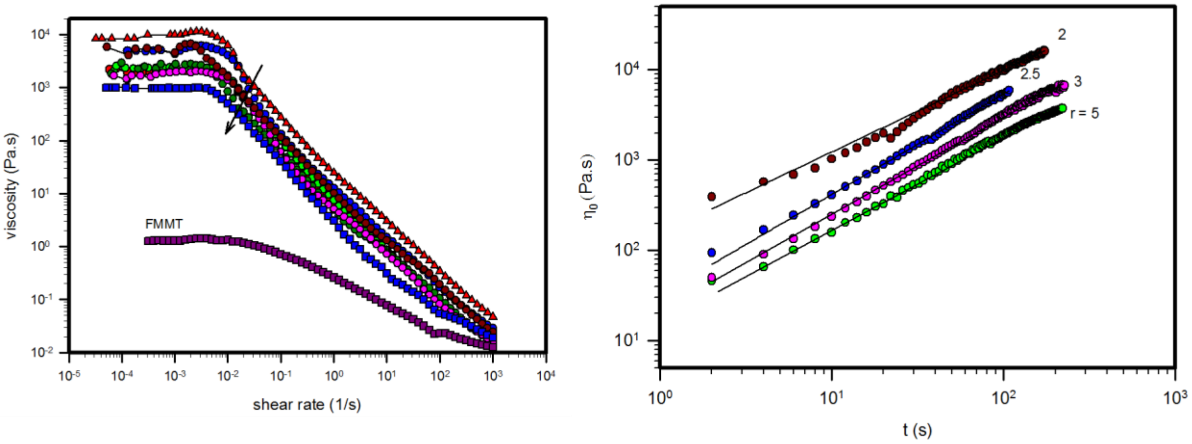

Figure S2 Flow curves and the zero-shear viscosity of the bicontinuous gels for different values of *r*.

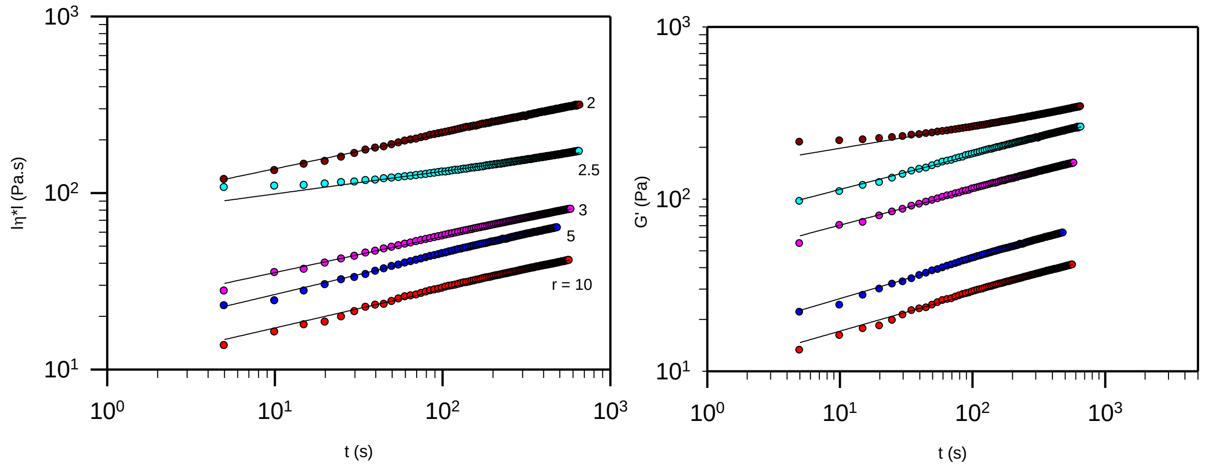

Figure S3. Growth of complex viscosity and elastic modulus with the waiting time at different values of *r*.

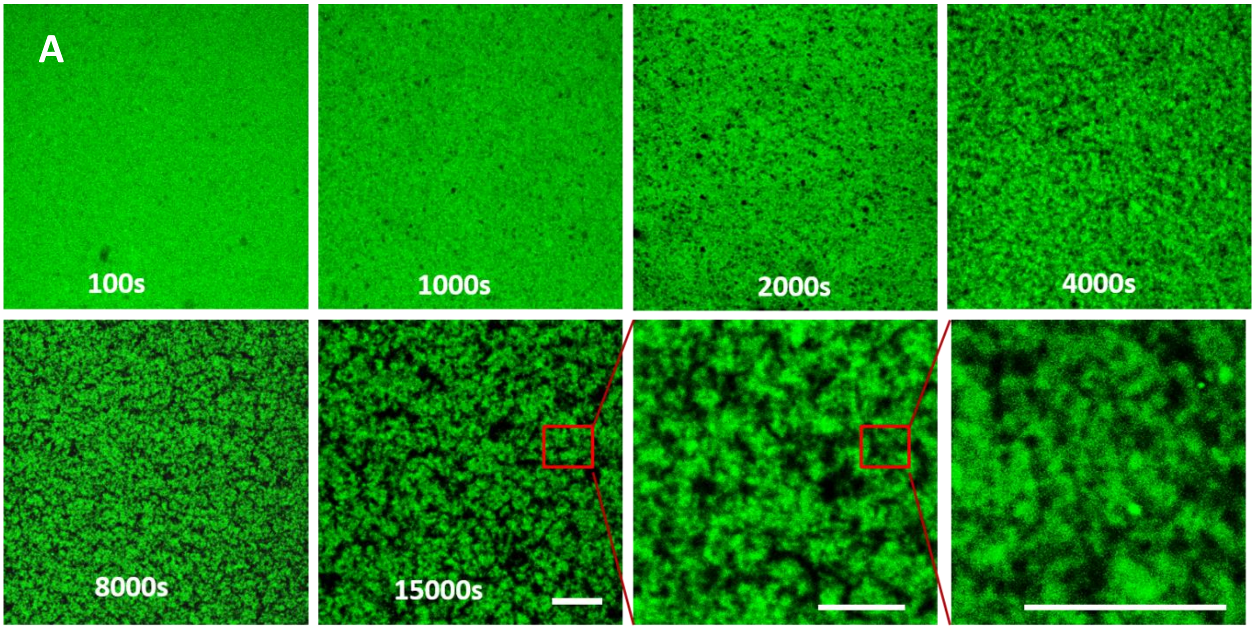

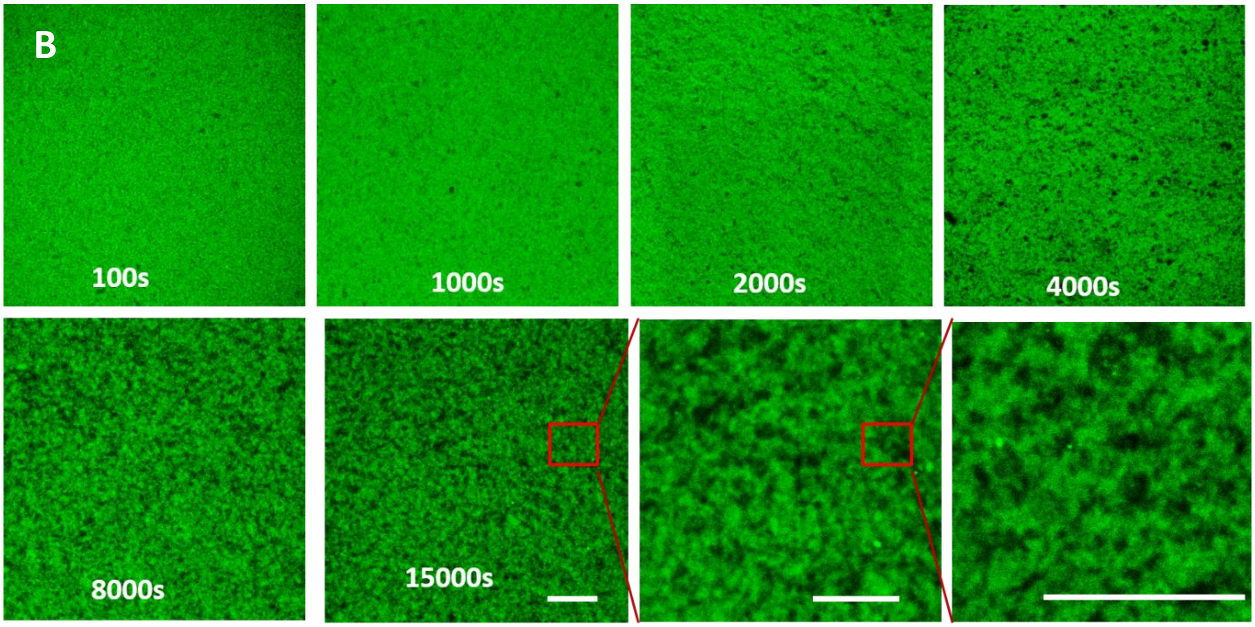

Figure S4 Evolution of gel structure with aging for *r* =*3* (A) and 2.5 (B). Scare bar is 20 μm.

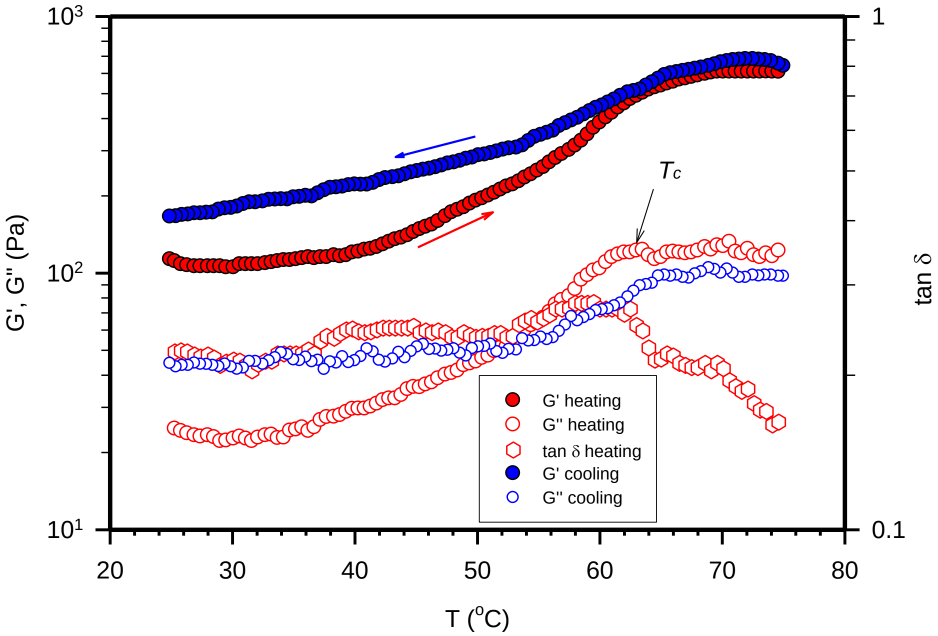

Figure S5. Temperature dependent rheological properties of the bicontinuous gel.

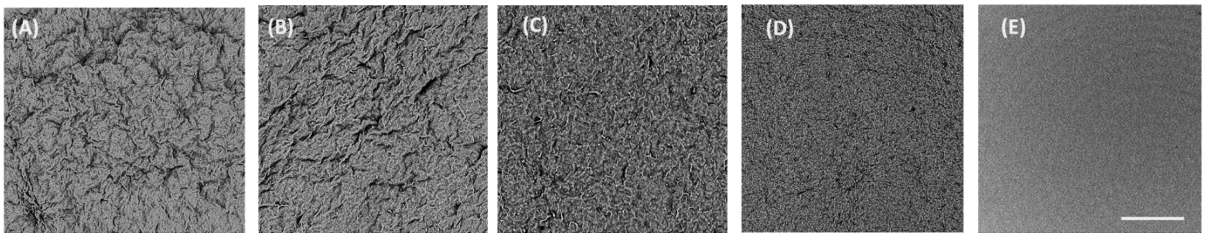

Figure S6 SEM images of dried films of FMMT, bicontinuous gels with ratios *r* = 8, 5, 3 and Laponite.

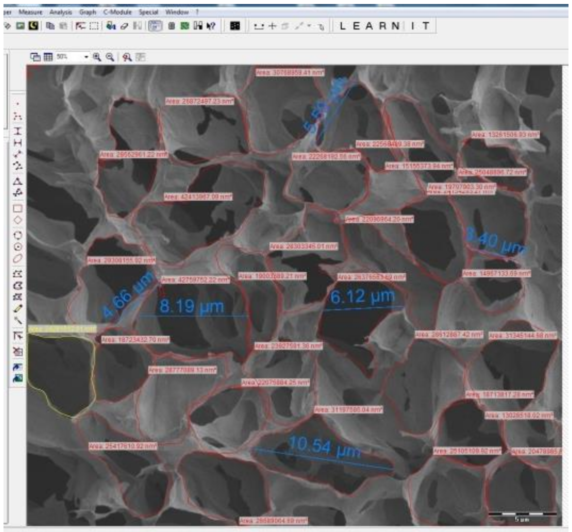

Figure S7 SEM analysis pore size and thickness of the branched of the BCG.

Clay	ρ (g/cc)	Aspect ratio (L/D)	CEC (meq/g)	D_{eff} (nm)	ζ (mV)	Physical state Concentration (c) dependent
**Laponite**	2.53	25	1.2	25	-40±2	c < 1 Phase Separation<br>1 < c < 2 Equilibrium Gels (Time Dependent)<br>2 < c < 3 Glass (Time Dependent)<br>c > 3 Repulsive/Attractive glass Nematic
**Na-MMT**	2.86	250	0.75	250	-32±2	c < 2.5 Phase Separation<br>2.5 < c < 5 Soft gels, Nematic<br>c > 5 Strong gels, Nematic
L+MMT					Linear combination	c < 0.3 Stable sol<br>0.3 < c < 2.2 Gel<br>c > 2.2 Glass, ordered glass

Table S1: Physio-chemical Properties of clays of Laponite and Na-MMT.

Time\sample	MMT (5%)	Lap (3%)	1:0.15	1:0.30	1:0.60	1:1	1:1.5
Day 1	43	40	53	55	57	63	57
Day 5	47	42	56	56	58	65	59
Day 10	49	45	58	58	61	67	60

Table S2: Transition temperatures of bicontinuous gels made from different mixing ratios.
